# Re-discovery and re-description of the true *Plagiopholisstyani* (Boulenger, 1899) (Serpentes, Pseudoxenodontidae), with the taxonomic status of the populations previously considered as *P.styani*

**DOI:** 10.3897/BDJ.13.e151488

**Published:** 2025-07-23

**Authors:** Shuo Liu, Zengyang Luo, Xi Xiao, Caichun Peng, Dongru Zhang, Shize Li

**Affiliations:** 1 Kunming Natural History Museum of Zoology, Kunming Institute of Zoology, Chinese Academy of Sciences, Kunming, China Kunming Natural History Museum of Zoology, Kunming Institute of Zoology, Chinese Academy of Sciences Kunming China; 2 Yunnan Key Laboratory of Biodiversity Information, Kunming Institute of Zoology, Chinese Academy of Sciences, Kunming, China Yunnan Key Laboratory of Biodiversity Information, Kunming Institute of Zoology, Chinese Academy of Sciences Kunming China; 3 Nanjian Management and Protection Bureau of Yunnan Wuliangshan National Nature Reserve, Nanjian, China Nanjian Management and Protection Bureau of Yunnan Wuliangshan National Nature Reserve Nanjian China; 4 Kuankuoshui National Nature Reserve Administration, Suiyang, China Kuankuoshui National Nature Reserve Administration Suiyang China; 5 Leigongshan National Nature Reserve Administration, Leishan, China Leigongshan National Nature Reserve Administration Leishan China; 6 School of Biological Science and Technology，Liupanshui Normal University, Liupanshui, China School of Biological Science and Technology，Liupanshui Normal University Liupanshui China; 7 Department of Food Science and Engineering, Moutai Institute, Renhuai, China Department of Food Science and Engineering, Moutai Institute Renhuai China

**Keywords:** cytb, distribution, morphology, Mountain Snake, systematics, taxonomy

## Abstract

**Background:**

The type locality of *Plagiopholisstyani* is in Wuyishan Mountain, Fujian Province, China and, currently, this species is considered to be widely distributed in southern China and northern Vietnam. However, since this species was described, there have been very few reports of this species from its type locality.

**New information:**

We re-discovered *Plagiopholisstyani* from its type locality and collected one topotypic specimen. By comparing the sequence of the topotypic specimen of *P.styani* with the sequences on GenBank, we found that the so-called sequences of *P.styani* on GenBank do not belong to *P.styani*. Based on the topotypic specimen of *P.styani*, we re-describe this species and provide molecular data of the true *P.styani* for the first time. Combining the sequences on GenBank and literature, we consider that the population from Sichuan and Guizhou Provinces, previously regarded as *P.styani*, to represent a new species of *Plagiopholis*. The taxonomic status of the populations previously considered as *P.styani* from other provinces of China and northern Vietnam still needs further evaluation.

## Introduction

The genus *Plagiopholis* Boulenger, 1893 is a rarely studied group of snakes, currently contains four valid species and all of which were described at least nearly a hundred years ago (Uetz et al. 2025). Although these species are widely distributed in southern China, northern Thailand, Myanmar, Laos and Vietnam (Das 2010), they are not easily encountered due to their secretive habits (Zhao 2006).

*Plagiopholisblakewayi* Boulenger, 1893 is the type species of the genus *Plagiopholis*. Its type locality is in Toungyi, Shan State, Myanmar (Zhao et al. 1998, Uetz et al. 2025). Currently, this species is considered to be distributed in Myanmar, Thailand and Yunnan and Guizhou Provinces of China (Zhao et al. 1998, Zhao 2006, Das 2010, Zhong et al. 2015, Huang 2021, Wang 2021, Uetz et al. 2025). At present, there are multiple gene sequences of this species in GenBank, all of which are from specimens collected in Yunnan, China.

*Plagiopholisdelacouri* Angel, 1929 was described from Xiengkhouang Province, which is located in north-eastern Laos and borders Vietnam (Orlov et al. 2003, Uetz et al. 2025). This species is currently known only in Laos and Vietnam (Das 2010, Zhong et al. 2015, Uetz et al. 2025). At present, there is no publicly available molecular data for this species.

*Plagiopholisnuchalis* (Boulenger, 1893) was also described from Toungyi, Shan State, Myanmar (Zhao et al. 1998, Uetz et al. 2025) and, currently, this species is considered to be distributed in Myanmar, Thailand and Yunnan Province of China (Zhao et al. 1998, Zhao 2006, Das 2010, Zhong et al. 2015, Wang 2021, Uetz et al. 2025). At present, there is also no publicly available molecular data for this species.

*Plagiopholisstyani* (Boulenger, 1899) was described from Kuatun, Fukien, which is located in Tongmu Village, Xingcun Town, Wuyishan City, Fujian Province, China. Afterwards, this species was found and recorded from Anhui (Zhao et al. 1998, Zhao 2006), Gansu (Zhao et al. 1998, Zhao 2006), Sichuan (Zhao et al. 1998, Zhao 2006), Zhejiang (Zhao et al. 1998, Zhao 2006), Guangxi (Zhao et al. 1998, Zhao 2006, Huang 2021, Wang 2021), Jiangxi (Zhao et al. 1998, Zhao 2006, Huang 2021, Wang 2021), Chongqing (Luo et al. 2004, Huang 2021), Hunan (Zhao 2006, Huang 2021, Wang 2021), Hubei (Dai et al. 2009), Taiwan (Xiang et al. 2009), Guizhou (Zhang et al. 2020) and Guandong (Huang 2021, Wang 2021) of China, as well as northern Vietnam (Orlov et al. 2003). However, this species has rarely been reported from its type locality again (Zhao et al. 1998). At present, there are some gene sequences of this species in GenBank, all of which are from specimens collected in Sichuan and Guizhou, China.

During our fieldwork in Fujian Province, China, in 2018, one specimen of *Plagiopholisstyani* was collected from its type locality; specific collection information can be found in the Taxon treatment section. Based on the topotypic specimen, we provide a re-description of this species. In addition, on the base of the genetic sequence of the topotypic specimen, we re-assess the taxonomic status of the population previously considered as *P.styani* from Sichuan and Guizhou Provinces herein.

## Materials and methods

The specimen was collected by hand; specific collection process and habitat information can be found in the Ecology notes section. After being photographed and euthanised using MS-222 solution (Simmons 2015), it was preserved in approximately 75% ethanol and then deposited at Kunming Natural History Museum of Zoology, Kunming Institute of Zoology, Chinese Academy of Sciences (KIZ) under the voucher KIZ20180002.

Total genomic DNA was extracted from liver tissue. A fragment of the mitochondrial cytochrome b gene (cytb) and a fragment of the cytochrome oxidase subunit I gene (COI) were amplified and sequenced using the primers L14910 (5’–GACCTGTGATMTGAAAACCAYCGTT–3’)/H16064 (5’–CTTTGGTTTACAAGAACAATGCTTTA–3’) (Burbrink et al. 2000) and Chmf4 (5’–TYTCWACWAAYCAYAAAGAYATCGG–3’)/Chmr4 (5’–ACYTCRGGRTGRCCRAARAATCA–3’) (Che et al. 2012). The amplification and sequencing were completed by Sangon Biotech (Shanghai) Co., Ltd. The new sequences have been deposited in GenBank and other sequences used in this study were obtained from GenBank (Table 1). *Pseudoxenodonmacrops* (Blyth, 1855) was used as outgroup according to Li et al. (2020).

Sequences were aligned using ClustalW (Thompson et al. 2002). Pairwise distances between species were calculated in MEGA 12.0.9 (Kumar et al. 2024). The best substitution models were selected under the Bayesian Information Criterion in ModelFinder (Kalyaanamoorthy et al. 2017). Bayesian Inference (BI) was performed in MrBayes 3.2.7 (Ronquist et al. 2012) using the HKY+F+G4 model for both cytb and COI and Maximum Likelihood (ML) analysis was performed in IQ-TREE 1.6.12 (Nguyen et al. 2015) using the TIM2+F+G4 model for both cytb and COI. The technical computation methods for BI and ML phylogenetic analyses were the same as those in Liu et al. (2024).

Measurements were taken with a ruler to the nearest 1 mm. Values for symmetric head characters are given in left/right order. Measurement and scale count methodology followed Liu et al. (2021): SVL, snout-vent length; TaL, tail length; DSR, dorsal scale rows, at one head length posterior to the head, at the mid-body, at one head length anterior to the vent, re­spectively; Lor, loreals; PreOc, preoculars; PostOc, postoculars; SL, supralabials; IL, infralabials; ATem, number of anterior temporals; PTem, number of posterior temporals; Prec, precloacal plate (divided or undivided); Ven, number of ventral scales; SubC, num­ber of subcaudal scales.

## Taxon treatments

### Plagiopholis styani

(Boulenger, 1899)

00F84A18-EB90-54FB-8977-1B2779042195

#### Materials

- **Type status:** Other material. **Occurrence:** catalogNumber: KIZ20180002; individualCount: 1; sex: female; lifeStage: adult; occurrenceID: EAA5E66E-45F0-519A-9027-AB6E53EFF7DF; **Location:** country: China; stateProvince: Fujian; locality: Guadun, Tongmu Village, Xingcun Town, Wuyishan City; verbatimElevation: 1550 m; verbatimCoordinates: 27°44′30″ N, 117°38′46″E; **Event:** eventRemarks: collected by Shuo Liu on 14 May 2018; **Record Level:** basisOfRecord: preserved specime

#### Description of the topotypic specimen

**Figure 1.**
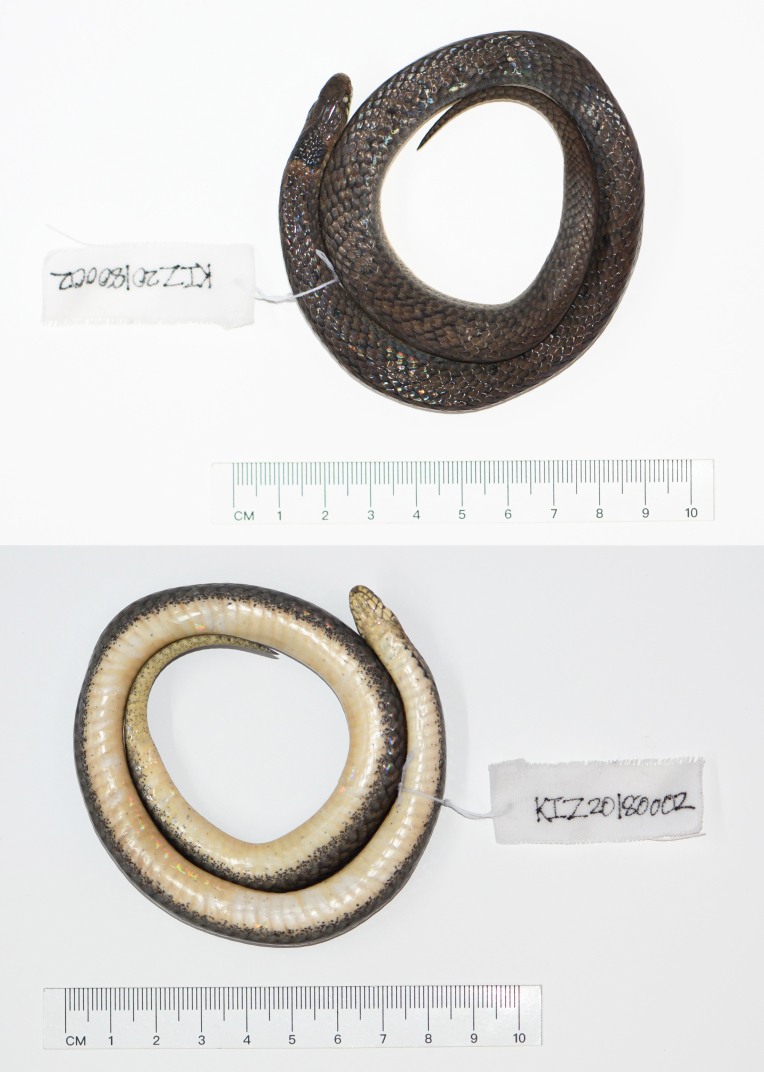
Dorsal view (top) and ventral view (bottom) of the topotypic specimen of *Plagiopholisstyani* in preservative.

**Figure 2.**
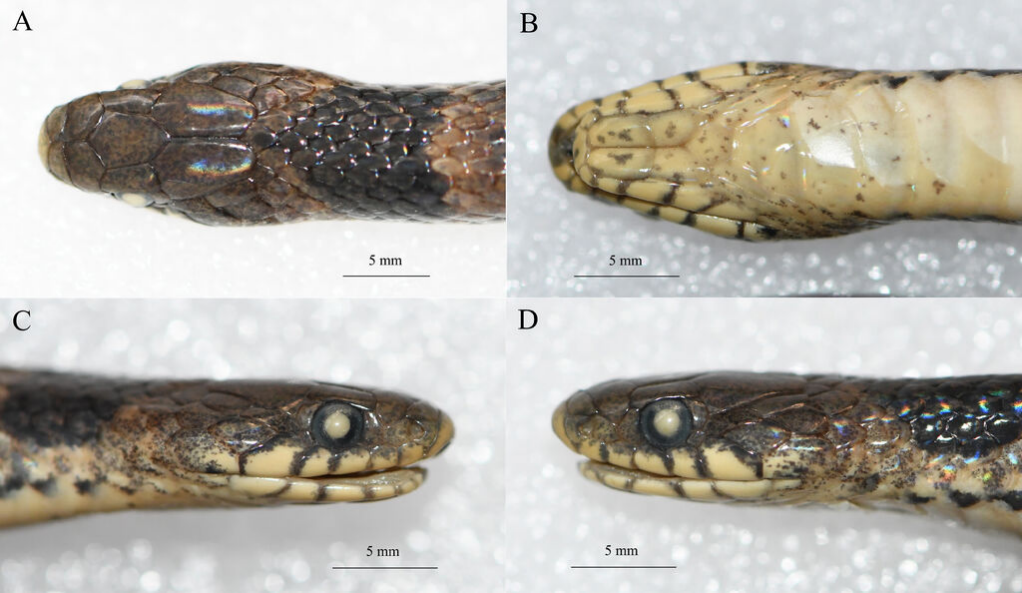
Close-up views of the head of the topotypic specimen (KIZ20180002) of *Plagiopholisstyani* in preservative. **A** dorsal views; **B** ventral view; **C** right view; **D** left view.

Adult female; body relatively short, tail quite short, SVL 359 mm, TaL 47 mm, TaL/SVL 0.13; head small, not distinct from neck; snout blunt, rostral large, approximately triangular, visible from above; internasals wider than long; prefrontals polygonal; frontal shield-shaped, longer than wide; supraocular elongated rectangular; parietals large, gradually narrowing posteriorly, median suture approximately equal to length of frontal; nasal divided into two scales; no loreal; eye moderate, pupil round; preocular 1/1; postoculars 2/2; supralabials 6/6, first and second in contact with prenasal and postnasal, third and fourth entering orbit; anterior temporals 2/2, posterior temporals 2/2; mental elongate, wider than long; infralabials 6/6, first pair not contacting each other; two pairs of chin shields, anterior pairs longer than posterior; dorsal scales in 15 rows throughout, all smooth; ventral scales 119; subcaudal scales 25; precloacal plate undivided (Table 2, Fig. 1, Fig. 2).

#### Colouration of the topotypic specimen in life

**Figure 3.**
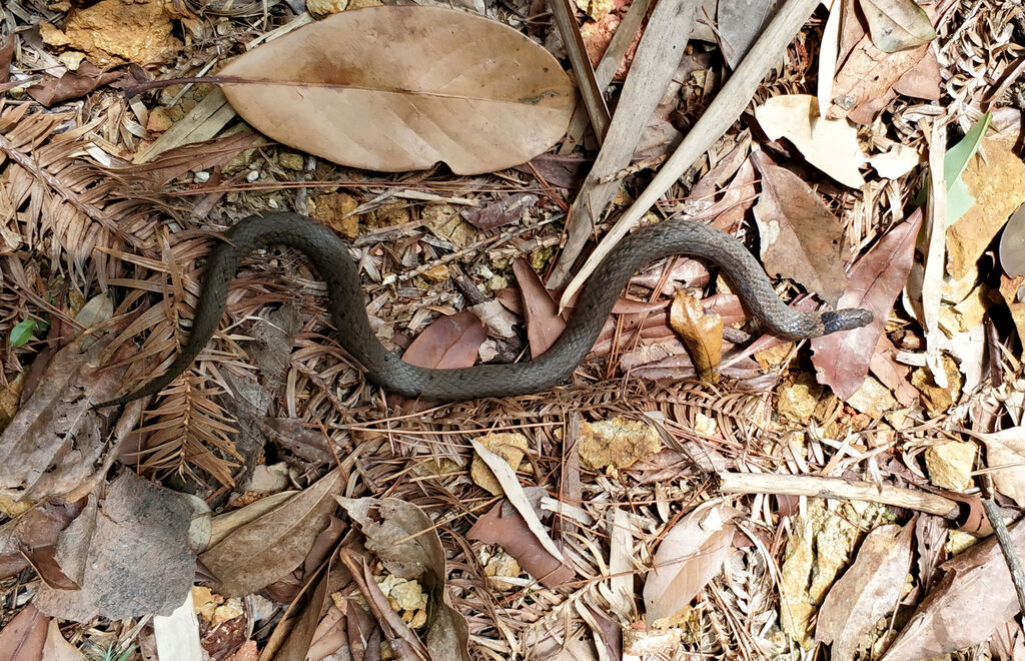
The topotypic specimen (KIZ20180002) of *Plagiopholisstyani* in life.

Dorsal surface greyish-brown with some small black spots on dorsum; a broad, approximately rectangular-shaped blotch on dorsal neck; region behind nuchal blotch slightly yellowish; labials light yellow with some vertical black stripes; iris light brown; ventral surface of head and body yellowish-white; ventral surface of tail light yellow with many tiny black spots (Fig. 3).

#### Distribution

**Figure 4.**
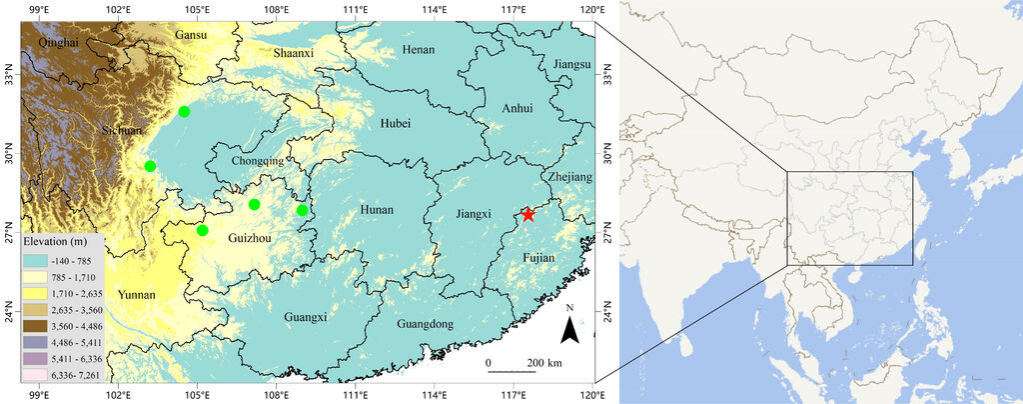
Map showing the type locality of *Plagiopholisstyani* in Fujian Province, China (red star) and the distribution of the potential new species of *Plagiopholis* in Sichuan and Guizhou Provinces, China (green dots). The elevation data were obtained from Geospatial Data Cloud (2024).

*Plagiopholisstyani* is currently confirmed to be only distributed in Wuyishan Mountain, Wuyishan City, Fujian Province, China (Fig. 4). It is speculated that it may also be distributed in nearby Zhejiang and Jiangxi Provinces.

#### Ecology notes

**Figure 5.**
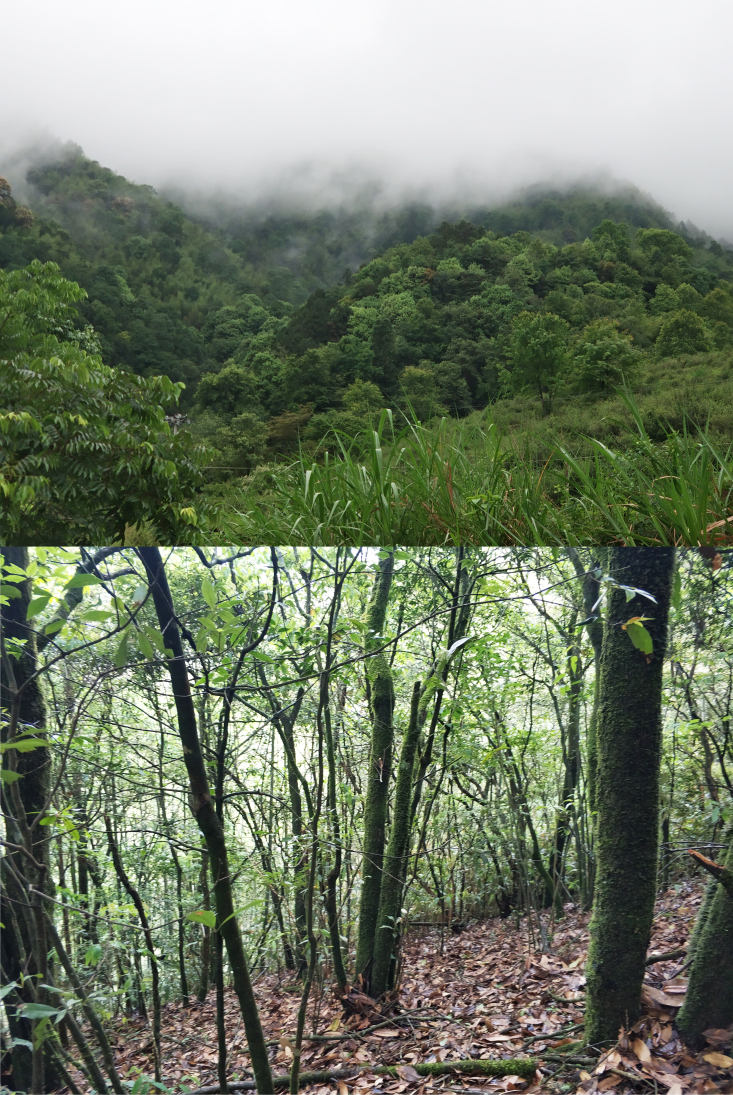
Distant view (top) and close view (bottom) of the habitat at the collection site of the topotypic specimen of *Plagiopholisstyani*.

The topotypic specimen was collected on the ground of a sunny hillside at approximately 10:00 a.m. The slope of the collection site is approximately 30°. The snake was hiding under the dead leaves and, when we passed by, it was disturbed and crawled out from under the dead leaves. The species is slow and easy to catch. No attack behaviour was observed. Nine other reptile species were found in the same habitats (Fig. 5) during the survey, namely *Acanthosauralepidogaster* (Cuvier, 1829), *Boigakraepelini* Stejneger, 1902, *Lycodonflavozonatus* (Pope, 1928), *Pareasformosensis* (Van Denburgh, 1909), *Protobothropsmucrosquamatus* (Cantor, 1839), *Pseudoxenodonstejnegeri* Barbour, 1908, *Sphenomorphusindicus* (Gray, 1853), *Viridoviperastejnegeri* (Schmidt, 1925) and *Xenochrophisflavipunctatus* (Hallowell, 1860).

## Analysis

**Figure 6.**
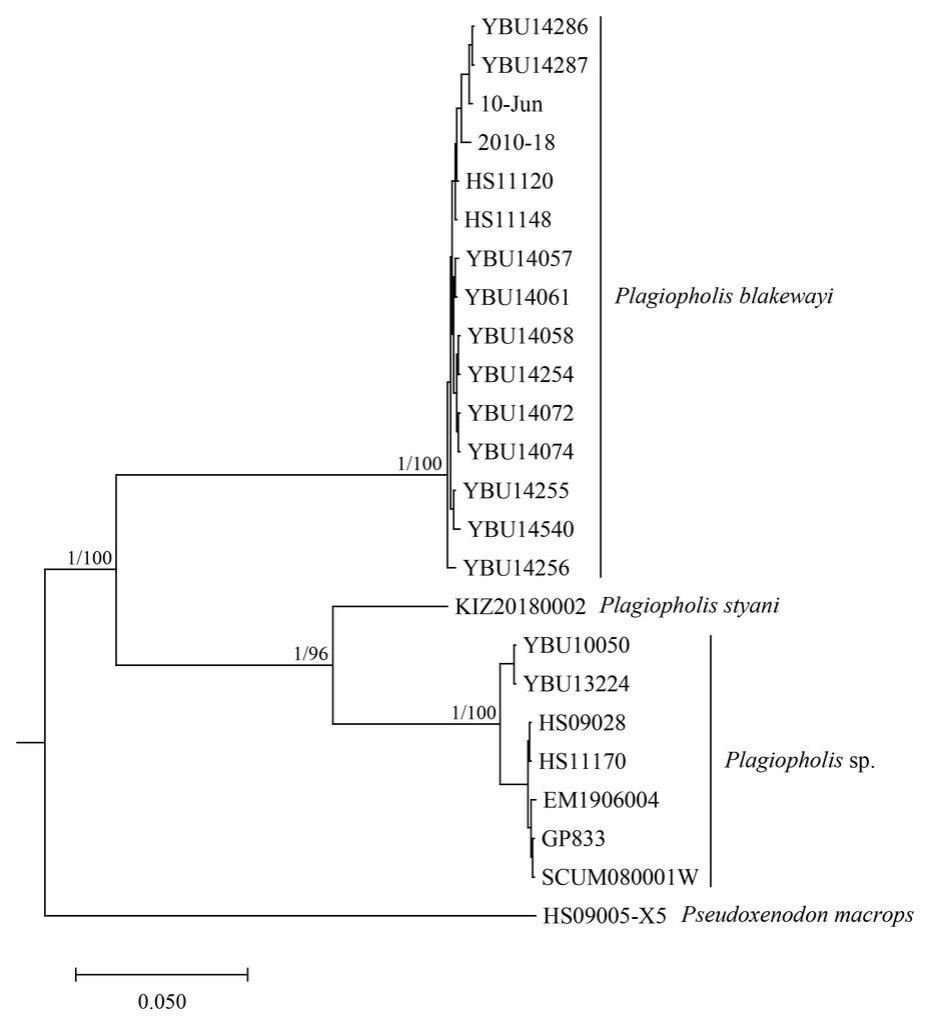
Bayesian phylogram of the genus *Plagiopholis* inferred from the conducted cytb and COI sequences. Numbers after and behind “/” are Bayesian posterior probabilities and ML ultrafast bootstrap values (values below 0.90/90 are not shown), respectively.

The BI and ML analyses yielded a consistent topology (Fig. 6). The sequence of the topotypic specimen of *Plagiopholisstyani* formed a distinct clade sister to the sequences of specimens from Sichuan and Guizhou Provinces, China, which were sourced from GenBank and considered to belong to *P.styani*, with strong support. The uncorrected pairwise distances between the sequence of the topotypic specimen of *P.styani* and the sequences that were considered to belong to *P.styani* from GenBank were 8.35% in cytb and 7.07% in COI (Table 3, Table 4). Therefore, we consider that the specimens previously regarded as *P.styani* from Sichuan and Guizhou Provinces do not belong to *P.styani*, but represent an undescribed species.

## Discussion

Since *Plagiopholisstyani* was described, there have been very few reports of this species from its type locality. Topotypic specimens are very important for species research, especially as they play an irreplaceable role in taxonomy. We re-discovered *P.styani* from its type locality and provided molecular data from a topotypic specimen of this species for the first time.

**Figure 7.**
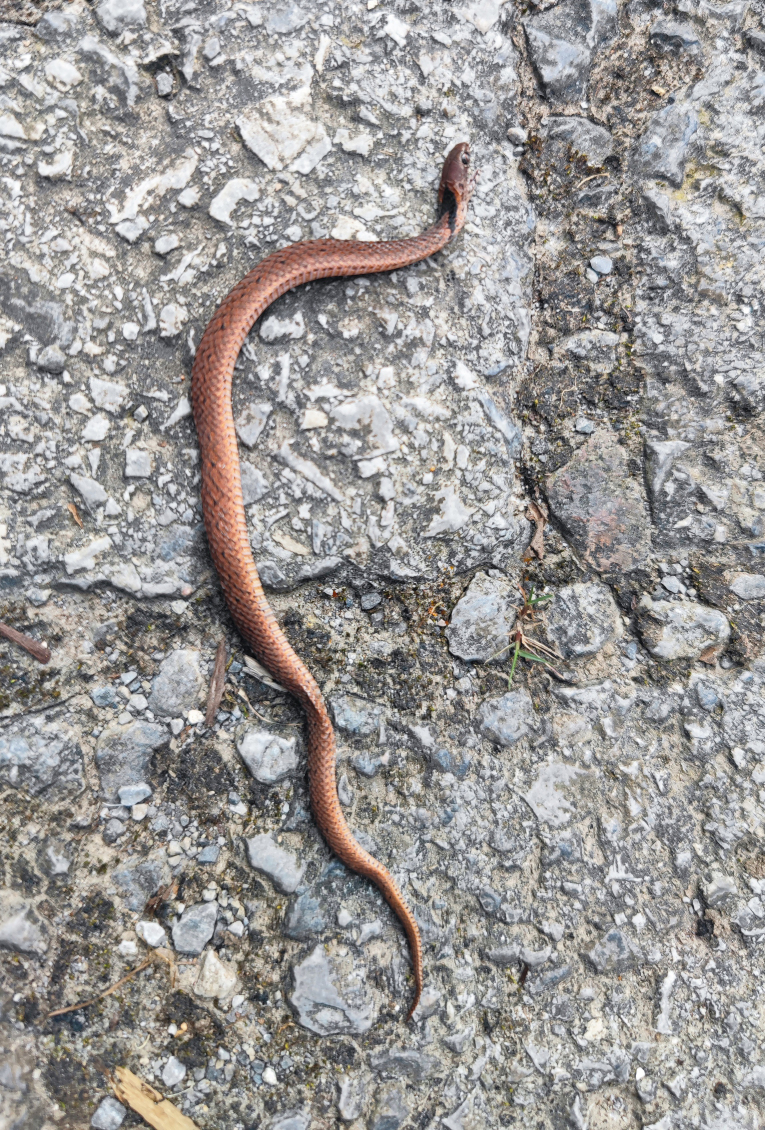
A roadkill of *Plagiopholis* from Guizhou Kuankuoshui National Nature Reserve, Suiyang County, Zunyi City, Guizhou Province, China.

Previously, *Plagiopholisstyani* was considered to be widely distributed in southern China (Huang 2021, Wang 2021). At present, there are some genetic sequences that are considered to belong to *P.styani* on GenBank, corresponding to specimens from Sichuan and Guizhou Provinces. Through phylogenetic analysis, we found that there was a significant genetic distance between these sequences of specimens from Sichuan and Guizhou and the newly-generated sequence of the topotypic specimen of *P.styani*. Morphologically, the topotypic specimen of *P.styani* agrees well with the original description of this species. In addition, the topotypic specimen is consistent with the illustrations of this species in the original publication as it has a broad, approximately rectangular-shaped nuchal blotch (Fig. 3). However, according to the photos in Zhao (2006) and Wang (2021) and our observation, the specimens from Sichuan and Guizhou have a narrow, distinctly V-shaped nuchal blotch (Fig. 7). Therefore, the true *P.styani* may only be distributed in Fujian and neighbouring Zhejiang and Jiangxi, while the population previously considered as *P.styani* from Sichuan and Guizhou represents an undescribed species of *Plagiopholis*, which we here refer to as *Plagiopholis* sp. As for the populations previously considered as *P.styani* from other provinces of China, as well as the population previously considered as *P.styani* from northern Vietnam, their taxonomic status still needs further evaluation as we have not obtained corresponding specimens or molecular data.

## Supplementary Material

XML Treatment for Plagiopholis styani

## Figures and Tables

**Table 1.** Samples used for the phylogenetic analyses in this study.

Species	Voucher	Locality	cytb	COI
*Plagiopholisblakewayi*	YBU14287	Mengzi, Yunnan, China	KT199012	/
*Plagiopholisblakewayi*	YBU14074	Mengzi, Yunnan, China	KT199005	/
*Plagiopholisblakewayi*	YBU14057	Mengzi, Yunnan, China	KT199009	/
*Plagiopholisblakewayi*	YBU14058	Mengzi, Yunnan, China	KT199010	/
*Plagiopholisblakewayi*	YBU14072	Mengzi, Yunnan, China	KT199004	/
*Plagiopholisblakewayi*	YBU14061	Mengzi, Yunnan, China	KT199003	/
*Plagiopholisblakewayi*	YBU14286	Mengzi, Yunnan, China	KT199011	/
*Plagiopholisblakewayi*	YBU14255	Mengzi, Yunnan, China	KT199007	/
*Plagiopholisblakewayi*	YBU14254	Mengzi, Yunnan, China	KT199006	/
*Plagiopholisblakewayi*	YBU14256	Mengzi, Yunnan, China	KT199008	/
*Plagiopholisblakewayi*	YBU14540	Mengzi, Yunnan, China	KT199013	/
*Plagiopholisblakewayi*	HS11120	Mengzi, Yunnan, China	MK201337	MK064682
*Plagiopholisblakewayi*	HS11148	Mengzi, Yunnan, China	MK201341	MK064686
*Plagiopholisblakewayi*	2010-18	Mengzi, Yunnan, China	MK201339	MK064684
*Plagiopholisblakewayi*	10-Jun	Yunnan, China	MK201340	MK064685
*Plagiopholisstyani*	KIZ20180002	Wuyishan, Fujian, China	PV847831	PV833807
“*Plagiopholisstyani*”	GP833	Mianyang, Sichuan, China	KT199000	/
“*Plagiopholisstyani*”	YBU10050	Tongren, Guizhou, China	KT199001	/
“*Plagiopholisstyani*”	YBU13224	Tongren, Guizhou, China	KT199002	/
“*Plagiopholisstyani*”	SCUM080001W	Mianyang, Sichuan, China	EU496918	/
“*Plagiopholisstyani*”	HS09028	Mianyang, Sichuan, China	MK201336	MK064681
“*Plagiopholisstyani*”	HS11170	Mianyang, Sichuan, China	MK201338	MK064683
“*Plagiopholisstyani*”	EM1906004	Leshan, Sichuan, China	MW697084	MW697084
*Pseudoxenodonmacrops*	HS09005-X5	Funiushan, Henan, China	MK201345	MK064690

**Table 2.** Measurements (in mm) and scalation data of the topotypic specimen of *Plagiopholisstyani*.

	KIZ20180002, ♀
SVL	359
TaL	47
DSR	15-15-15
Lor	0/0
PreOc	1/1
PostOc	2/2
SL	6 (2-2-2)/6 (2-2-2)
IL	6/6
ATem	2/2
PTem	2/2
Prec	undivided
Ven	119
SubC	25

**Table 3.** Uncorrected pairwise genetic distances (%), based on cytb sequences.

	*Plagiopholisstyani*	*Plagiopholisblakewayi*	*Plagiopholis* sp.
*Plagiopholisstyani*	/		
*Plagiopholisblakewayi*	13.94	0.21	
*Plagiopholis* sp.	8.35	14.14	0.57

**Table 4.** Uncorrected pairwise genetic distances (%), based on COI sequences.

	*Plagiopholisstyani*	*Plagiopholisblakewayi*	*Plagiopholis* sp.
*Plagiopholisstyani*	/		
*Plagiopholisblakewayi*	13.05	0.18	
*Plagiopholis* sp.	7.07	13.79	0.10
